# Determinants and Assembly Mechanism of Bacterial Community Structure in Ningxia Section of the Yellow River

**DOI:** 10.3390/microorganisms11020496

**Published:** 2023-02-16

**Authors:** Rui-Zhi Zhao, Wei-Jiang Zhang, Zeng-Feng Zhao, Xiao-Cong Qiu

**Affiliations:** 1 School of Civil and Hydraulic Engineering, Ningxia University, Yinchuan 750021, China; 2 School of Life Science, Ningxia University, Yinchuan 750021, China

**Keywords:** Ningxia section of the Yellow River, bacterial community structure, co-occurrence patterns network, driving factors, assembly mechanism

## Abstract

The Yellow River is a valuable resource in the Ningxia Hui Autonomous Region and plays a vital role in local human activities and biodiversity. Bacteria are a crucial component of river ecosystems, but the driving factors and assembly mechanisms of bacterial community structure in this region remain unclear. Herein, we documented the bacterial community composition, determinants, co-occurrence pattern, and assembly mechanism for surface water and sediment. In comparison to sediment, the bacterioplankton community showed significant seasonal variation, as well as less diversity and abundance. The network topology parameters indicated that the sediment bacterial network was more stable than water, but the bacterioplankton network had higher connectivity. In this lotic ecosystem, COD_Mn_, Chl *a*, and pH affected the structure of the bacterioplankton community, while TP was the primary factor influencing the structure of the sediment bacterial community. The combined results of the neutral community model and the phylogenetic null model indicate that Bacterial communities in both habitats were mainly affected by stochastic processes, with ecological processes dominated by ecological drift for bacterioplankton and dispersal limitation for sediment bacteria. These results provide essential insights into future research on microbial ecology, environmental monitoring, and classified management in the Ningxia section of the Yellow River.

## 1. Introduction

Rivers are among Earth’s most dynamic, diverse, and complex ecosystems [1]. Rivers and their tributaries form a vertical continuum called a river network, a complex organization linking water, land, and sea [2]. Rivers contribute to the biodiversity of many landscapes [3]. At the same time, they provide irreplaceable service functions for nature and society, determining biodiversity, ecosystem productivity, and human health and wellbeing in the region [4]. Nevertheless, the ecological functions of river networks are not static, and in the context of climate change and increased human activity, the ecological functions of some rivers have already been affected [5,6,7,8], the ecological functions they are burdened with need to be re-evaluated as soon as possible. Bacteria are considered to be an essential component of riverine ecosystems, not only as contributors to nutrient and energy flows [9,10], but also as significant players in the biogeochemical processes and ecosystem functions of river networks [11]. In addition, changes in bacterial community structure are closely related to the environment. Bacterial diversity can be used as an indicator of river pollution [12]. To a certain extent, the function of river ecosystems is determined by microbial functional diversity [13]. Therefore, the investigation of bacterial community structure and functional characteristics, spatial and temporal dynamics, and their association with the environment will not only help to understand the current status of river ecosystems, but also provide theoretical support for ecological conservation and restoration of water ecology.

In the last 20 years, high-throughput sequencing technology has changed our understanding of the bacterial community by establishing a new area and providing a practical method for microbial community study [14,15]. Revolutionary advances in sequencing technologies can more easily reveal the microbial composition of environmental samples, thus contributing to the comprehension of the role played by microorganisms in the aquatic ecosystems, especially in rivers [16,17].

The Yellow River, the second-largest river in China, originates at the northern foot of the Bayankara Mountains on the Qinghai-Tibet Plateau and empties into the Bohai Sea. It is also the world’s largest turbid river, with a multi-year (1960~2020) average sand content of 20.13 g/L [18]. High turbidity creates an environment with limited light, which inhibits the growth of primary products like phytoplankton and photosynthetic bacteria [19,20]. Additionally, the turbulent environment strongly alters the structure of the riverbed and microclimate, scouring, and siltation, which stresses river species [21,22]. Recently, several studies have reported on the bacterial diversity, spatiotemporal distribution, assembly mechanisms, and environmental interactions in the Yellow River basin. For instance, in the middle reaches of the Yellow River, including from Xiaolangdi Reservoir to Kaifeng Bridge, the dominant phylum of planktonic bacteria is *Proteobacteria*, with bacterial diversity increasing along the lower reaches of the river [23]. Similar dominant communities are common to the turbid mainstem and clear tributaries of the Weihe River, one of the important tributaries in the middle reaches of the Yellow River, and geographical distance has a significant effect on the structure of the bacterioplankton community [24]. Furthermore, *α-Proteobacteria* and *β-Proteobacteria* are the dominant phylum in the overlying waters of the Yellow River estuary and show extremely high sensitivity to environmental factors [25]. However, these studies have tended to focus on the middle and lower reaches and estuaries. There is still a lack of reference studies reported in the upper Yellow River.

Ningxia Hui Autonomous Region is located in the upper reaches of the Yellow River. In this region, the Yellow River provides the most important water source for agriculture, industry, and population. Therefore, the water ecological health of the Ningxia section of the Yellow River is vital to the sustainable development of the Ningxia region. However, the natural conditions of its ecosystems have been negatively impacted by solid sediment transport, climate change, and anthropogenic disturbances. The current state of the water ecology needs to be comprehensively investigated and assessed. Some recent studies have reported on the bacterial community in the Ningxia section of the Yellow River [26,27]. Nevertheless, they are all based on a large-scale sampling of the entire Yellow River mainstem, and no in-depth surveys have been carried out on small-scale river sections by setting up more sampling points. In addition, the fragmented findings make it difficult to provide a holistic picture of the complexity and variability of the bacterial communities in the Ningxia section of the Yellow River.

In this study, the bacterial community structure of 48 samples (including water bodies and surface sediments) from three seasons in the Ningxia section of the Yellow River was studied using high-throughput sequencing technology. Our research goals were: (1) analyze the composition of the bacterial community structure in different habitats and its distribution pattern; (2) identify the main environmental parameters that influence the composition of the bacterial community in different habitats; and (3) reveal the dominant processes that affect the assembly of microbial communities in two habitats. The study results will provide critical information for assessing and managing aquatic ecosystems in the Ningxia section of the Yellow River, and serve as a scientific basis for restoring the river’s water ecology and biodiversity conservation.

## 2. Materials and Methods

### 2.1. Study Area, Sample Collection, and Chemical Analysis

**Figure 1:**
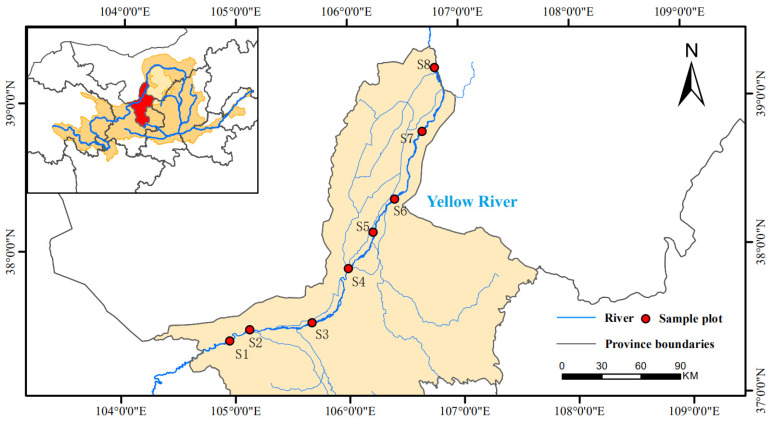
Distribution of sampling sites in the Ningxia section of the Yellow River.

The Ningxia section of the Yellow River flows from Nanchangtan in Zhongwei City to Mahuanggou in Shizuishan City, covering 397 km and accounting for 7% of the Yellow River’s entire length. In addition, the Qingshui River, the Kushui River, the Hongliu Ditch, and many other artificial drainage ditches are injected into the Ningxia section of the Yellow River, forming the Ningxia Yellow River Basin. The watershed ranges from 35°50′~39°23′ N and 104°17′~107°12′ E, covering an area of 41,600 km^2^, accounting for 81% of the total area of Ningxia. In accordance with the project requirements, eight sampling points were set up in the mainstream of the Ningxia section of the Yellow River (Figure 1), and samples were taken in April, July, and October 2021.

Three parallel samples (2 L each) at 50 cm depth were randomly collected at the sampling point and mixed as water samples (6 L). After labeling, the water sample was placed in a polyethylene sampling bottle and stored in an incubator below 4 °C before being transported to the laboratory. The water samples for DNA analysis were filtered through 0.22 μm filter membrane within 24 h (under sterile conditions). The filter membranes were stored at −80 °C until DNA extraction. The sample pretreatment process was carried out in a sterile environment to avoid the contamination of samples by external bacteria. Sediment samples were collected using a modified Peterson mud collector. Three parallel surface sediment samples (sampling depth: 10 cm) were randomly collected from each sample site. After mixing, the samples were packed in a sealed plastic bag, immediately placed in a 4 °C incubator, and transported back to the laboratory for processing and analysis.

Water temperature (WT), electrical conductivity (Cond), salinity (Sal), dissolved oxygen (DO), pH, total dissolved solids (TDS), and chloridion (Cl^−^) were all measured using a YSI Pro-plus portable water quality analyzer. Fluoride (F^−^) was measured on site using a HACH HQ40d portable water quality analyzer. Chlorophyll a (Chl *a*) was measured on site using a HACH Hydrolab DS5X. Total nitrogen (TN), total phosphorus (TP), available phosphorus (AP), ammonia nitrogen (NH_4_^+^-N), nitrite nitrogen (NO_2_^−^-N), permanganate index (COD_Mn_), and chemical oxygen demand (COD_Cr_) were determined according to the method provided by the *Water and Wastewater Detection and Analysis Method (4th edition) (Ministry of Ecology and Environment of the People’s Republic of China 2002)*. Sulfate (SO_4_^2−^) was tested according to *the water quality determination of the sulfate-Gravimetric method (GB 11899-89)*. Soil organic matter (SOM) was tested according to the *method for determination of soil organic matter (GB 9834-88)*. Based on the previous method [28], arsenic (As), chromium (Cr), mercury (Hg), and lead (Pb) concentrations were determined by inductively coupled plasma mass spectrometry (ICP-MS).

### 2.2. Extraction, High-Throughput Sequencing, and Bioinformatics Analysis

Total DNA was extracted using NucleoSpin 96 soi (MACHEREYNAGEL, Dueren, Germany) following the manufacturer’s protocol. DNA concentration and purity were monitored on 1% agarose gels. According to the concentration, DNA was diluted to l ug/μL using sterile water, and 16S rRNA genes of V3-V4 regions were amplified using specific primers 341F (5′-CCTACGGGAGGCAGCAG-3′) and 806R (5′-GGACTACHVGGGTWTCTAAT-3′) with the barcode. All PCR reactions were carried out with 15 μL of Phusion^®^ High-Fidelity PCR Master Mix (New England Biolabs, Ipswich, MA, USA), 0.2 μm of forward and reverse primers, and around 10 ng template DNA. Thermal cycling consisted of initial denaturation at 98 °C for 1 min, followed by 30 cycles of denaturation at 98 °C for 10 s, annealing at 50 °C for 30 s, and elongation at 72 °C for 30 s. Finally, at 72 °C for 5 min.

After mixing the PCR product with the IX loading buffer (SYB green), electrophoresis was performed on 2% agarose gel. PCR products were mixed in equidensity ratios. Then, mixture PCR products were purified using the Qiagen Gel Extraction Kit (Qiagen, Hilden, Germany). Sequencing libraries were generated using the TruSeq^®^ DNA PCR-Free Sample Preparation Kit (Illumina, San Diego, CA, USA). Following manufacturer’s recommendations, sequencing libraries were generated using the NEBNext^®^ Ultra™ IIDNA Library Prep Kit (Cat No. E7645). After the library quality at Novogene Technology Co., Ltd. (Tianjin, China). Purified amplicons were sequenced using the strategies of PE250 on an Illumina NovaSeq 6000 platform.

The platform QIIME2 (v2021.4, https://qiime2.org, accessed on 21 April 2022) was used to process and analyze the raw fastq files [29]. Quality controls, annotations, statistical calculations, and diversity analyses were implemented using the standard QIIME2 Pipeline. Denoise was performed with DADA2 to obtain initial ASVs (Amplicon Sequence Variants), and then ASVs with an abundance less than 0.01% were filtered out [30]. After obtaining the feature table, the taxonomic classification was processed using the Silva reference database (Release 138.1, http://www.arb-silva.de, accessed on 27 July 2022) [31].

### 2.3. Statistical Analysis

Due to the non-normal distribution of the data in this study, the Kruskal–Wallis test was used for difference analysis of alpha diversity, bacterial community composition, and environmental parameters; whereas the Dunn test (corrected by the Bonferroni method) was used for multiple comparisons. This process was completed in R by the package “rstatix” (version 0.7) [32]. An analysis of the differences in habitats and sampling periods in the Ningxia section of the Yellow River was conducted using nonmetric multidimensional scales (NMDS) based on Bray Curtis distances. The R-value obtained from the Analysis of Similarity (ANOSIM) was used to quantify the degree of difference within the bacterioplankton community. The larger the R-value, the higher the degree of difference between groups. This analysis was completed using the R “vegan” package (version 2.6-2) [33]. Specificity-occupancy (SPEC-OCCU) plots were used to inidentify of potential keystone species in water and sediment; SPEC-OCCU plots were drawn using the “ggpolt2” package (version 3.4.0) [34]. The comparison of phyla between the two habitats was completed using STAMP software (version 2.1.3).

The variance inflation factor (VIF) was calculated to check the collinearities, and variables with VIF > 20 were removed from the analysis to avoid the impact of cross-collinearity. The forward selection was used to select variables driving bacterial community composition using the “ordiR2step” function from vegan (version 2.6-2), all nonsignificant (*p* > 0.05) variables were eliminated in further analyses [35]. Except for pH, the environmental parameters were log (x + 1) transformed to satisfy the normality and homogeneity requirements. For network analysis, only genera with a relative abundance greater than 0.005 in each habitat and occurring at more than five sample sites were retained to construct co-occurrence pattern networks, and the paired Spearman correlation was calculated using R ”Hmisc” package (version 4.4.0) [36]. The correlation coefficient matrix was formed using the correlation coefficient ≥ |0.7| with a *p*-value less than 0.05 (Benjamini and Hochberg adjusted) as the standard. The co-occurrence pattern network was visualized using Cytoscape (version 3.9.0) [37]. In addition, the differences between the co-occurrence pattern networks were captured by the dissimilarity index (β_w_) [38].

The beta nearest taxon index (βNTI) was calculated to measure the relative contribution of different assembly processes. A significant deviation (|βNTI| > 2) indicated the dominance of selection processes (deterministic processes) during the succession of the community. Among these, βNTI > 2 represented variable selection, while βNTI < –2 represented homogeneous selection. On the contrary, |βNTI| < 2 meant that the stochastic process was important in community assembly. The non-significant βNTI value (|βNTI|< 2) was calculated using Raup-Crick (RC bray) based on Bray-Curtis. Generally, RC_bray_ < −0.95 is defined as homogeneous dispersal, RC_bray_ > 0.95 represents dispersal limit, and |RC_bray_| ≤ 0.95 represents drift [39,40]. The above process was calculated using the R “iCAMP” package (version 1.5.12) [41]. Moreover, to identify the potential contribution of stochastic processes to the assembly of bacterial communities in the Ningxia section of the Yellow River, the NCM (neutral community model) was used to correlate the observed occurrence frequency of ASVs with mean relative abundance [42,43]. The parameter R^2^ was used to indicate the overall fit to the neutral model; R^2^ > 0 means that the population conforms to the neutral model (stochastic processes), while R^2^ < 0 indicates the opposite. The m-value represents the immigration rate. It was uniform for each community member (independent of species), with smaller m-values indicating more restricted species dispersal throughout the community. Conversely, higher m-values indicated less restricted species dispersal. The calculation and plotting of NCM were accomplished by using packages “Hmisc” (version 1.8.2) and “minpack.lm” (version 1.2.2) in R.

## 3. Results

### 3.1. Diversity of Bacteria in the Ningxia Section of the Yellow River

After quality control and filtration, the 48 samples from the Ningxia section of the Yellow River generated a total of 2,548,101 high-quality reads, with an average of 54,035 per sample. Those reads were assigned to 1854 ASVs in the water and 4405 ASVs in the sediment. The rarefaction curves, ACE and Chao1 indices indicated that the sample sequencing amount was sufficient, and the sequencing depth covered most of the species in the sample (Figure S1, Table S1).

**Figure 2:**
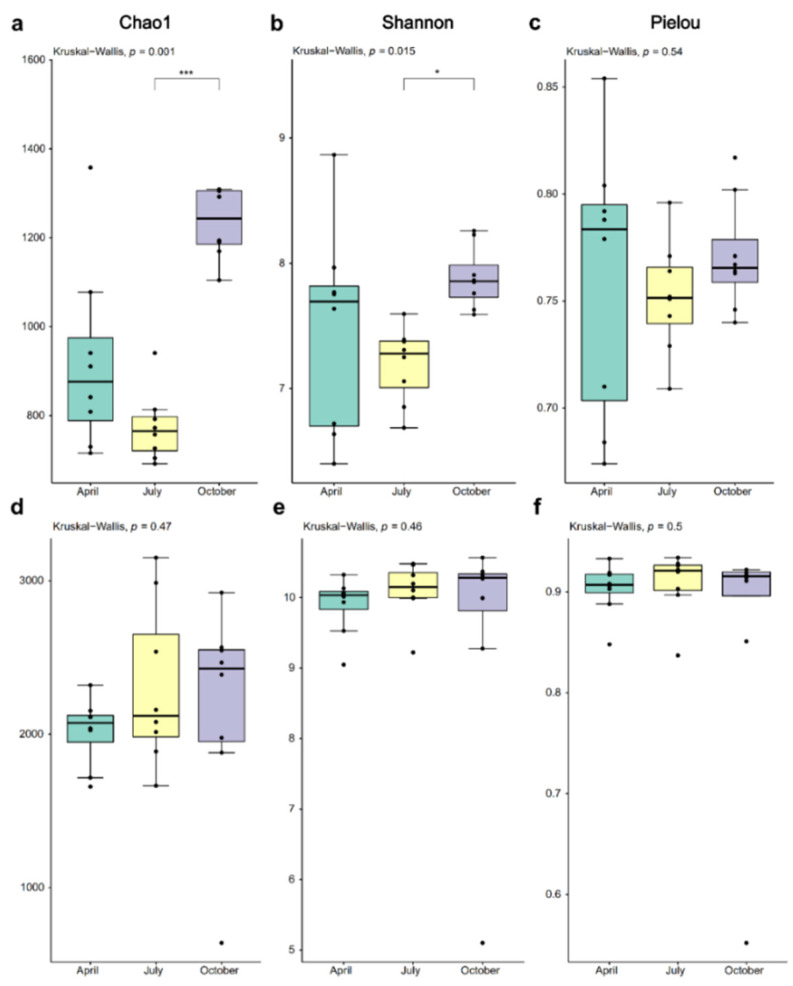
Comparison of alpha diversity indexes between sampling periods in two habitats with the Kruskal-Wallis test and the Dunn test ((**a**–**c**), water; (**d**–**f**), sediment). *, *p* < 0.05, *** *p* < 0.001.

The Kruskal-Wallis test results for diversity indicators of bacterioplankton community, as shown in Figure 2a–c, demonstrated that the differences in Chao1 and Shannon index among the three sampling periods were statistically significant, while the Pielou’s evenness index was not (*p* = 0.001 and *p* = 0.015, respectively). October’s Chao1 and Shannon index were higher than the other two sampling periods. Furthermore, Dunn-test (Bonferroni correction) results showed that those two diversity indicators significantly differed between October and July. The Pielou’s evenness index was higher in April, but there were no significant differences between groups (*p* = 0.54). However, the diversity indexes showed a different pattern in the sediment samples. All of them revealed no significant variations among the three sampling periods (Chao1, *p* = 0.47; Shannon, *p* = 0.46; Pielou, *p* = 0.50; Figure 2d–f). The complete alpha diversity indexes are summarized in Table S1.

### 3.2. Community Composition and Dynamics of Bacteria in the Two Habitats

**Figure 3:**
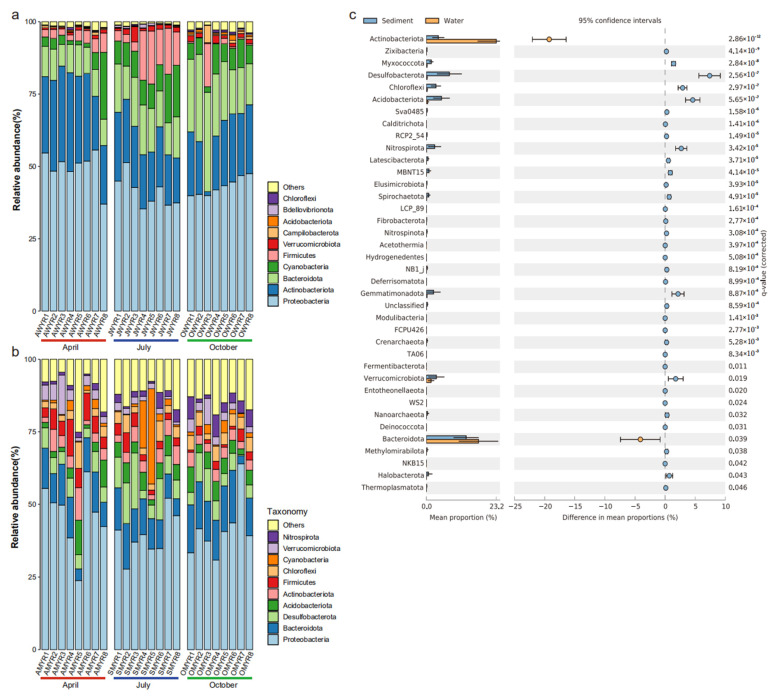
Relative abundances of the top 10 bacterial phyla in the two habitats ((**a**) water; (**b**) sediment). The remaining phyla were defined as “other”. The extended error bar plot (**c**) shows multiple clades with differences in the two habitats. The left bar represents the number of ASVs in each clade, the middle is the 95% confidence interval, and the right is the corrected *p*-value.

*Proteobacteria* (44.66%, average relative abundance), *Actinobacteriota* (22.16%), *Bacteroidota* (15.35%), *Cyanobacteria* (7.60%), *Firmicutes* (5.57%), *Verrucomicrobiota* (1.36%), *Campilobacterota* (0.59%), *Acidobacteriota* (0.37%), *Bdellovibrionota* (0.31%), and *Chloroflexi* (0.28%) were the major phyla of the whole bacterioplankton community (57 phyla) in the Ningxia section of the Yellow River (Figure 3a). Different from bacterioplankton, the bacterial community in the sediment samples included 77 phyla. The primary phyla were *Proteobacteria* (42.20%), *Bacteroidota* (11.98%), *Desulfobacterota* (7.07%), *Acidobacteriota* (4.88%), *Actinobacteriota* (4.18%), *Firmicutes* (4.06%), *Chloroflexi* (3.78%), *Cyanobacteria* (3.39%), *Verrucomicrobiota* (3.28%), and *Nitrospirota* (2.71%) (Figure 3b). A total of 37 differential phyla were screened by Welch’s *t*-test in both habitats. Figure 3c demonstrated that, with the exception of *Actinobacteriota* and *Bacteroidota*, the abundance of each differential phylum was higher in sediment than in water.

As shown in Figure S2a, except for *Chloroflexi*, the relative abundance of the other phyla significantly varied over the three sampling periods in water. However, only the *Firmicutes*, *Verrucomicrobiota*, and *Nitrospirota* significantly differed over the sample periods in sediment (Figure S2b). The variations in bacterial community composition between the two types of samples were more easily observed at the family level. For instance, more dominant families of *Bacteroidota* were found in water (Figure S3). In contrast, the dominant families of *Proteobacteria* were more numerous in the sediment samples (Figure S4). Moreover, seasonal transitions have been observed between families within the same phylum. However, the pattern of variation is different for each family.

The most abundant 500 ASVs in each habitat were selected as the dominant ASVs. To examine how dominant ASVs were distributed across habitats and how specific they were to a habitat, the occupancy and specificity of these ASVs were then calculated and projected onto the map. ASVs with specificity and occupancy greater than or equal to 0.7 were identified as specialist species (specific to a habitat and shared in their habitat in most sites).

**Figure 4:**
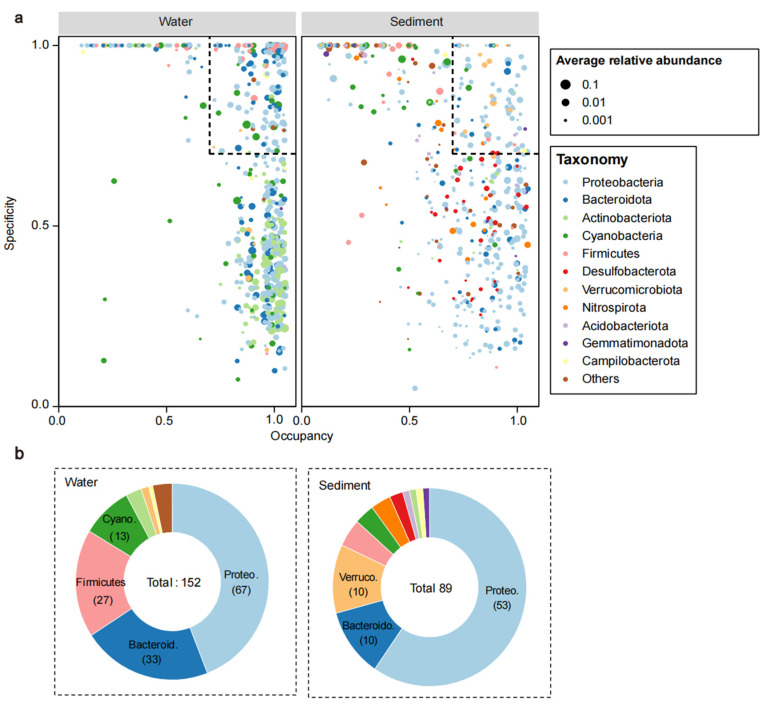
The SPEC-OCCU plots (**a**) show 500 most abundant ASVs in each habitat, the x-axis indicates the occupancy rate of ASVs in all sample sites, the y-axis indicates the specificity of ASVs in the habitat. The dotted box shows the specialized species (specificity and occupancy greater or equal to 0.7). Pie charts (**b**) show the number of ASVs representing specialists in each habitat.

According to Figure 4a, the ASVs from water samples exhibited homogeneous occupancy, yet the ASVs from sediment communities exhibited different occupancy at each site. Furthermore, a total of 241 specialist ASVs were found in the two habitats, of which 152 belonged to water samples and 89 to sediment. *Proteobacteria*, *Bacteroidota*, Actinobacteriota, *Cyanobacteria*, *Firmicutes*, *Verrucomicrobiota*, and *Campilobacterota* were found in both habitat specialist groups, but differed in the number of ASVs. In addition, sediment specialists included ASVs from *Desulfobacterota*, *Nitrospirota*, *Acidobacteriota*, and *Gemmatimonadota*. In addition, we also identified the dominant ASVs at the family level for different sampling periods in each habitat. Detailed results are presented in Figures S5 and S6.

**Figure 5:**
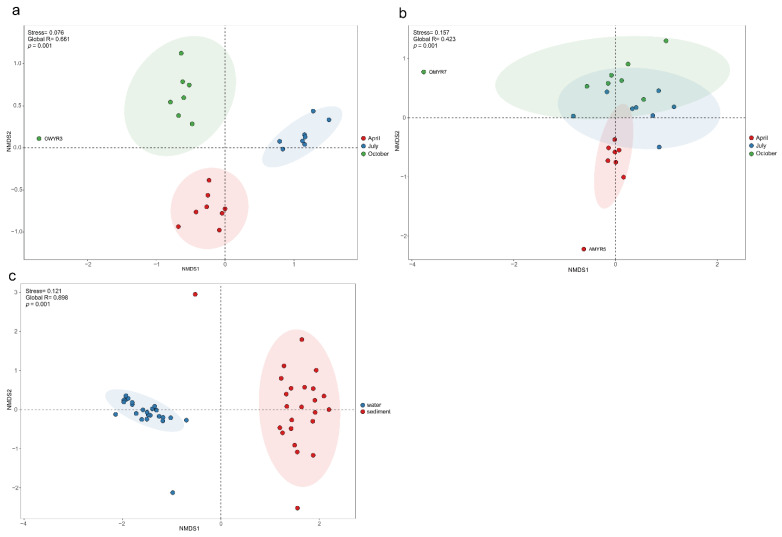
NMDS (Nonmetric multidimensional scaling analysis) and ANOSIM for bacterioplankton communities in sampling periods (**a**,**b**) and habitats (**c**) on Bray–Curtis similarity. Stress < 0.2 provides a good representation in NMDS. Global R > 0 means the grouping is valid. Moreover, *p* < 0.05 indicates a significant difference. Ellipses are 95% confidence intervals around the centroid.

The NMDS based on the Bray-Curtis distance was used to evaluate the bacterial community composition. The result in Figure 5a show that the community of bacterioplankton have a prominent seasonal group (Global R = 0.661, *p* = 0.001). In contrast, the seasonal groups were not evident in the sediment bacterial community for bacterioplankton (Global R = 0.423, *p* = 0.001, Figure 5b). Furthermore, bacterial community composition was extremely different in the two habitats (Global R = 0.898, *p* = 0.001, Figure 5c).

### 3.3. The Major Environmental Drivers of Bacterial Community

A total of 26 environmental parameters were chosen for this study, including 16 environmental factors for water and 10 for sediments (Table 1). According to the Kruskal-Wallis test for water environmental parameters, WT, pH, DO, Chl *a*, TN, NH^4+^-N, TP, and COD_Mn_ were substantially different (*p* < 0.05) among the three sampling periods, while other parameters were not significantly different. In the sediments, the other parameters’ seasonal differences were insignificant, except for OM, TN, AP, and As, which showed significant seasonal variation (*p* < 0.05).

After environmental variables (VIF > 20) were excluded, forward selection (ordiR2step, 999 permutations) was used to determine the drivers of bacterial community composition. The results suggested that COD_Mn_, pH, Chl *a*, and DO significantly (*p* < 0.05) revealed variation in bacterioplankton community composition; whereas the variation in bacterial community composition in the sediment was significantly (*p* < 0.05) explained by TP. The VIF values for each environmental variable are shown in the Table S2.

Co-occurrence pattern networks between the dominant bacterial genera (total relative abundance > 0.5% and occurring at more than five sample sites) and environmental variables were established for the two habitats, respectively. In the water samples, the network consisted of 200 nodes and 1722 edges, with a diameter of 9, a density of 0.087, a modularity index of 0.323, an average path length of 3.138, an average clustering coefficient of 0.551, and an average path length of 3.138 (Table 2). In the sediment samples, the network consisted with 276 nodes and 658 edges, with a diameter of 14, a density of 0.017, a modularity index of 0.708, an average path length of 5.531, an average clustering coefficient of 0.373, and an average path length of 5.531 (Table 2). The two networks are significantly different (β_w_ = 1), indicating that the bacterioplankton network has stronger small-world properties than the sediment bacterial network.

**Figure 6:**
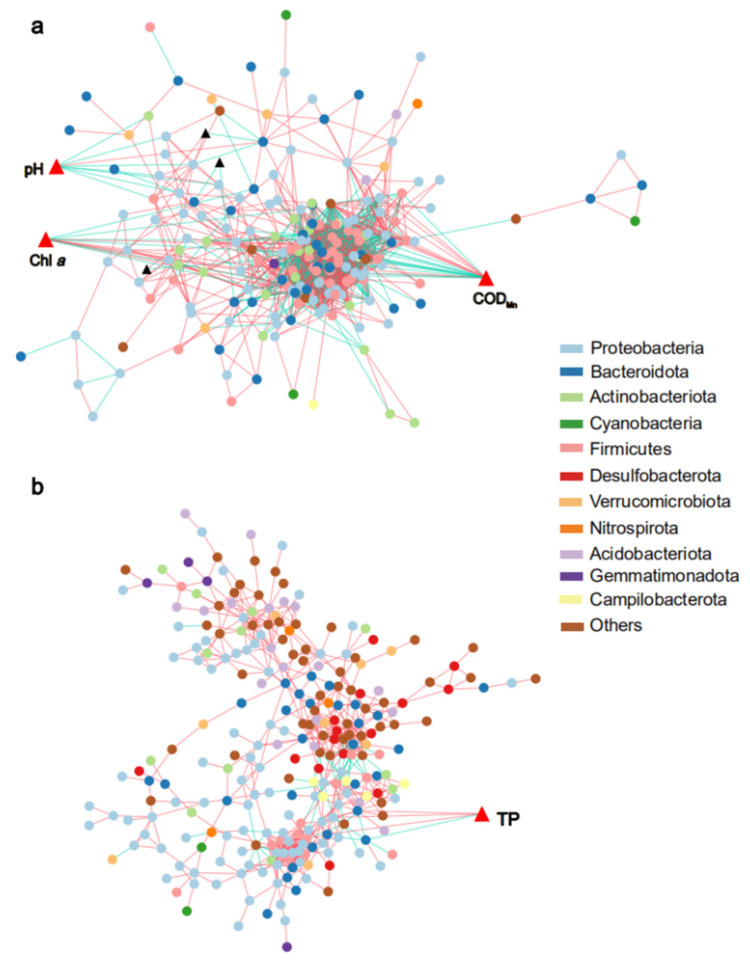
The co-occurrence pattern network shows the connectivity among bacterial communities and environmental parameters ((**a**) water; (**b**) sediment). The circle nodes represent different genera, the triangle represents different environmental factors involved in network construction, while red triangle represents main factors. A blue solid line signifies a positive correlation, and a red solid line signifies a negative correlation. COD_Mn_ represents the permanganate index, Chl *a* represents chlorophyll a, and TP represents total phosphorus.

In the bacterioplankton network, COD_Mn_, Chl *a*, and pH showed higher associations with major genera than other environmental variables. When the connections between the nodes were examined, it was observed that the genera connected to COD_Mn_ and subject to positive correlation were primarily from the *Comamonadaceae* and *Methylophilaceae* (both belong to *Proteobacteria*), while the negatively correlated genera were mainly from *Lachnospiraceae* and *Ruminococcaceae* (*Firmicutes*). The genera from *Comamonadaceae*, *Moraxellaceae*, *Microbacteriaceae*, *Carnobacteriaceae*, and *Chitinophagaceae* were positively connected to Chl *a*. The first two belong to *Proteobacteria* and the last three belong to *Actinobacteriota*, *Firmicutes*, and *Bacteroidota*, respectively. The vast majority of bacteria negatively associated with Chl *a* were from *Proteobacteria*. Fewer neighbors were connected to the pH node; only the *Spirosomaceae* (*Bacteroidota*) were negatively correlated with pH, and most of the positively correlated genera belonged to *Proteobacteria* (Figure 6a). In contrast, most environmental factors in the sediments were not connected to their neighbors, and only TP was connected to a few edges. Among them, *Muribaculaceae* (*Bacteroidota*) was negatively correlated with TP. *Cyclobacteriaceae* and *Porphyromonadaceae* (both belong to *Bacteroidota*), and *Rhizobiaceae* (*Proteobacteria*) were positively correlated with TP (Figure 6b). The taxonomic orders of bacteria and their correlations with major environmental factors were shown in Table S3.

### 3.4. Ecological Assembly Mechanism of Bacterial Community in Different Habitats

**Figure 7:**
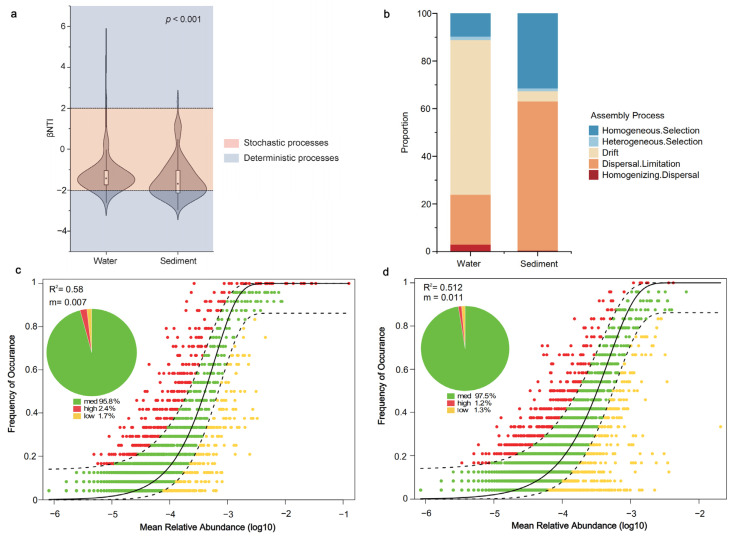
The neutral community model (NCM) and the phylogenetic null model for bacterial communities in the Ningxia section of the Yellow River. (**a**) Distribution of beta nearest taxon index (βNTI), |βNTI|< 2 indicated a stochastic process, |βNTI|> 2 indicated a deterministic process; (**b**) Relative contributions of deterministic and stochastic processes evaluated by null model framework; (**c**) Fit of the neutral community model (NCM) based on bacterioplankton community; (**d**) Fit of the neutral community model (NCM) based on sedimentary bacteria community. The ASVs represented by red dots showed a higher frequency of occurrence than the model predicts, while those represented by yellow dots were the opposite. The ASVs predicted by the 95% confidence interval of the model are shown as green dots.

To further assess the relative contributions of deterministic and stochastic processes to the formation of bacterial communities in the Ningxia section of the Yellow River. The bacterial community assembly mechanisms were examined by using the neutral community model (NCM) and the phylogenetic null model. Following the results in Figure 7, the neutral community model predicted most relationships between ASV occurrence frequency and their relative abundance change. The model’s interpretation rate (R^2^) suggested that stochastic processes had a strong effect on bacterial community assembly in both habitats, accounting for 0.580 and 0.512 of the bacterial community variation in water and sediments, respectively (Figure 7c,d).

Meanwhile, the results of the null model also showed the dominant effect of stochastic processes on bacterial community assembly mechanisms (most |βNTI| < 2, Figure 7a). From Figure 7b, it was shown that, in the bacterioplankton community (88.77%), the proportion being dominated by stochastic processed was higher than those of the sedimentary bacteria community (67.39%). Ecological drift (64.86%) was the dominant process for bacterioplankton community assembly, while sedimentary bacterial community assembly was primarily influenced by dispersal limitations (62.68%).

## 4. Discussion

In this study, 48 samples were collected from the surface water and sediment of the Ningxia section of the Yellow River, with 24 samples obtained from each habitat. We characterized the bacterial communities and determined the factors affecting their structure. Our results revealed significant differences in bacterial composition, diversity, symbiotic patterns, dominant drivers, and assembly processes between the two habitats.

The Chao1 index, Shannon index, and Pioule index were used to characterize bacterial communities’ abundance, diversity, and evenness. The results showed that the indices of sediment bacterial communities in the Ningxia section of the Yellow River were all higher than those of bacterioplankton communities, consistent with the global pattern of microbial diversity [44]. The bacterial community in surface water was found to be more dynamic compared to the sediment bacterial community, indicating that it was more susceptible to environmental influences [45]. The α-diversity index in this study was calculated based on ASVs, which precludes comparison with studies utilizing OTUs as their clustering units. However, large-scale ecological patterns should remain robust regardless of feature clustering methods [46]. For instance, our study found that the dominant phyla in the Ningxia section of the Yellow River had similarities with the results from two previous studies that utilized OTUs [27,28]. However, they were not identical, especially regarding their relative abundance.

Analysis of the bacterial community composition in the Ningxia section of the Yellow River revealed that the sum of the relative abundance of the dominant bacteria (relative abundance >1%) in the bacterioplankton community exceeded 96.70%, and the most dominant taxa were *Proteobacteria*, followed by *Actinobacteriota* and *Bacteroidota*. *Cyanobacteria*, *Firmicutes*, and *Verrucomicrobiota* were also the dominant phyla of bacterioplankton. Our findings were similar to previous studies in other rivers [24,47] and consistent with the bacterial community composition of typical freshwater bodies [48]. In the sediments, the dominant phyla were *Proteobacteria*, *Bacteroidota*, *Desulfobacterota*, *Acidobacteriota*, *Actinobacteriota*, *Firmicutes*, *Chloroflexi*, *Cyanobacteria*, *Verrucomicrobiota*, and *Nitrospirota*. Studies in freshwater lakes [49] and rivers [50] have found that *Proteobacteria* was the most abundant phylum in all sediment samples and was involved in the functioning and processing of freshwater sediment ecosystems [51], such as the inhibition of pathogenic microorganisms, nitrogen, and phosphorus transformation [52]. To probe the specific differences in patterns of bacterial communities between the two habitats, we used STAMP to conduct further analysis. The results indicated a dominance in the number of ASVs belonging to sediment bacteria across most phyla. A recent study confirmed that the high bacterial diversity of sediments is partly due to their ability to recruit and subsequently deposit microbes from surrounding sources, such as soil, sand, and plant debris [53]. Moreover, the sediment provides a stable environment, which can support the growth and reproduction of bacteria. Certainly, the sediment in the Ningxia section of the Yellow River serves as a hub for material cycling and energy transfer. An abundant number of species maximize ecological niche opportunities and enables better energy transfer and nutrient utilization [54]. Nevertheless, it is noteworthy to contemplate the transformation of sediment bacteria and bacterioplankton under strong current disturbance in this turbid, lotic ecosystem.

In this study, NMDS analysis (Figure 5c) clearly separated bacterial communities, and we also identified the occupancy and specificity of the different ASVs (Figure 4). This suggested that habitat type was the main reason for the differences in community structure. Undoubtedly, water and sediment are two different habitats with different environmental conditions, such as the amount of organic matter and nutrient content, all of which affect the composition of the bacterial community [55]. In addition, there was a significant seasonal separation of bacterial communities in the Ningxia section of the Yellow River, and this phenomenon was more significant in the bacterioplankton community (Globe R_water_ > Globe R_sediment_, Figure 5a,b). The Kruskal-Wallis test results (Figure S2a) showed seasonal differences in the major phyla in each habitat. In the water, there were significant differences in the relative abundance of the nine major phyla, except for *Chloroflexi*. However, in the sediments, the differences between seasons were only reflected in the three phyla (*Fimicutes*, *Verrucomicobiota*, and *Nitrospirota*) with lower relative abundances. In fact, some environmental parameters (e.g., WT, pH, and TN) in water did considerably vary among the sampling periods (Table 1), and their significant seasonal variations strongly influenced the composition of the bacterioplankton community [56]. Strong perturbations by hydrological factors have caused increased environmental stresses, leading to changes in the bacterioplankton community [57]. The gentle topography and slow water flow in the Ningxia section of the Yellow River result in less disturbance of the sediments. The sediment environment reaches equilibrium through long-term sediment erosion and accumulation, so the seasonal changes in the sediment bacterial community are less significant than those of bacterioplankton [58].

Co-occurrence pattern network analysis helps to decipher complex microbial community structures [59], while the topological parameters are a direct representation of network properties, and they can also be used for network aggregation and connectivity assessment [56,60]. In this study, we separately constructed co-occurrence pattern networks for the bacterial communities in the two habitats. Although the number of nodes in the bacterioplankton network was lower than that in the sediment, there was a clear numerical advantage in edges (connections). It was indicated that a higher contact frequency of the bacterioplankton community. The higher average clustering coefficient, average path lengths and density demonstrated that the complexity of the bacterioplankton co-occurrence network was higher than that of sedimentary bacteria. Additionally, there was a tight association among the major genera. Modules are groups of nodes that are well connected with one another, but less connected with nodes belonging to other modules [43,61]; nodes in the same module occupy similar ecological functions and niches [62]. Higher modularity indicated that the sediment bacterial community had more ecological niches and could maintain community stability through functional and ecological niche concentration, thus minimizing environmental impacts [63].

Bacterial community structure is assumed to result from external environmental factors and the random distribution of different habitats [64]. Multiple external environmental factors may affect bacterial community structure for a lotic system, such as the Yellow River [65]. Our study showed that the influencing parameters of bacterioplankton and sediment bacteria in the Ningxia section of the Yellow River were very different. After removing environmental factors with high covariance (VIF > 20), COD_Mn_, pH, Chl *a*, and DO had significant effects on the composition of the bacterioplankton (*p <* 0.05), TP was significantly associated with sediment bacteria (*p <* 0.05). COD_Mn_ is a conventional measure of the contamination by organic and oxidizable inorganic matter in a water sample. In previous studies, the bacterioplankton community structure varies depending on chemical oxygen demand levels [66]. It is widely recognized that pH is essential to bacterial community structure. pH not only directly affects bacterial growth status, but also influences bacterial community structure and diversity by changing the physicochemical properties of water [67]. Chl *a* is an important indicator of phytoplankton extant, the metabolism of heterotrophic bacteria is closely related to phytoplankton. Meanwhile, both metabolites and secretions of phytoplankton could affect the bacterial community composition, and Chl *a* content indirectly influences the density changes of planktonic bacteria in the water body [68]. Aerobic bacteria need oxygen to grow and develop due to the lack of antioxidant enzymes, while anaerobic bacteria need to live in an anoxic environment. Therefore, DO is also a primary factor in developing the bacterioplankton community structure [69]. In addition, TP is one of the determinants of bacterial community structure in sediments, which could change bacteria’s overall structure and function by affecting denitrifying bacteria [70,71]. The co-occurrence networks revealed that COD_Mn_, Chl *a*, and pH were associated with multiple nodes in the bacterioplankton network, and a few dominant genera were associated with TP in another network. Combining the results of the two analyses, we suggest that COD_Mn_, Chl *a*, and pH mainly drove the bacterioplankton community in this study. However, the main driver factor of sediment bacteria community was TP.

In recent years, the in-depth mechanisms of community assembly have been one of the central challenges and hotspots in microbial ecology. Substantial evidence has shown that deterministic and stochastic processes play a crucial role in the turnover of riverine microbial communities [43,72], and many studies have focused on their different importance [73,74]. In the present study, the effect of ecological processes on bacterial communities in two habitats was first evaluated using a phylogenetic null model [39,40]. Our findings unequivocally demonstrate that stochastic processes play a significant role in assembling bacterial communities in the Ningxia section of the Yellow River. In comparison to the bacterial community in the sediment, the effect of stochastic processes on the assemblage of the bacterioplankton community was more significant (Figure 4a). Furthermore, the dominance of ecological drift in the bacterioplankton community and dispersal limitation in sediment bacterial communities were confirmed by quantifying different ecological processes in community assembly (Figure 4b). Indeed, the NCM results (Figure 4c,d) reiterated the importance of stochastic processes in the two habitats. Our study on the mechanism of bacterioplankton community assembly was in agreement with the results of other rivers, where the stochastic process (dispersal limitation) was the primary process shaping the communities [47,75]. Nevertheless, the findings of this study conflict with those of other studies regarding sediment bacteria. For instance, Yuan et al. [76] found that homogeneous selection was the dominant ecological process for bacterial community assembly in large river sediment. When Lu et al. [77] studied the bacterial communities in estuarine sediments, they discovered that although stochastic processes had a significant impact on bacterial community assembly, deterministic processes were more crucial. Wang et al. [78] also pointed out that the main ecological process determining the bacterial community compositions in water was the dispersal process, whereas in sediments it was the selection process. The discrepancy in the study results may be attributed to the small environmental gradients and spatiotemporal scales in the present study area. In addition, the slower change in elevation gradient may also be a reason [73].

## 5. Conclusions

The examination of bacterial communities is an important part of monitoring environmental and ecological conditions in rivers. In this study, we examined the bacterial community structure, environmental drivers, and potential assembly mechanisms in two habitats (water column and sediment) in the Ningxia section of the Yellow River. There were some differences in the community structure and diversity between bacterioplankton and sedimentary bacteria. Significant seasonal variation was observed in the bacterioplankton community, but not in the sediment bacterial community. The results showed that the environmental variables COD_Mn_, Chl *a*, and pH affected the structure of the bacterioplankton community, while TP was the primary factor influencing the structure of the sediment bacterial community. Bacterial communities in both habitats were mainly affected by stochastic processes, with ecological processes dominated by ecological drift for bacterioplankton and dispersal limitations for sediment bacteria. It is noteworthy that the seasonal cycle in this area is an annually recurring process, and whether the dynamics of the bacterial community are similarly recurring in the future needs to be researched in the long term. Future research should pay more attention to the effects of shifting environmental gradients because the process of microbial community assembly is not static. In addition, many natural tributaries and artificial channels converge into the mainstream of the Yellow River to form a complex river network. The development and assembly mechanisms of bacterial communities in this system are still unexplored and should also be encouraged in future studies.

## Figures and Tables

**Table 1:** Water environment parameters (mean ± SD) of the Taiyangshan wetland in different sampling periods.

Habitat Type	Enviromental Parameters	April	July	October	Statistical Significance
Water	WT (°C)	14.19 ± 0.96	18.14 ± 1.24	10.84 ± 1.73	***
	pH	7.17 ± 0.19	8.00 ± 0.05	8.27 ± 0.17	***
	DO (mg/L)	7.89 ± 0.60	7.05 ± 1.07	6.25 ± 0.41	***
	Cond (μS/cm)	510.68 ± 99.44	575.19 ± 146.53	540.00 ± 140.10	ns
	Sal (ppt)	0.31 ± 0.06	0.34 ± 0.07	0.28 ± 0.08	ns
	TDS (mg/L)	416.57 ± 73.10	428.08 ± 101.53	379.00 ± 98.41	ns
	Chl *a* (mg/L)	18.68 ± 7.41	5.21 ± 0.98	8.47 ± 5.32	***
	TN (mg/L)	1.89 ± 0.32	2.23 ± 0.34	0.48 ± 0.12	***
	NH_4_^+^-N (mg/L)	0.26 ± 0.17	0.15 ± 0.13	0.76 ± 0.38	***
	TP (mg/L)	0.03 ± 0.02	0.06 ± 0.02	0.07 ± 0.04	*
	AP (mg/L)	0.06 ± 0.06	0.05 ± 0.05	0.04 ± 0.03	ns
	COD_Mn_ (mg/L)	1.38 ± 0.35	0.99 ± 0.15	2.08 ± 0.32	***
	COD_Cr_ (mg/L)	9.41 ± 4.45	7.88 ± 2.35	10.51 ± 2.23	ns
	Cl^−^ (mg/L)	100.08 ± 57.3	70.06 ± 25.32	112.69 ± 37.19	ns
	F^−^ (mg/L)	0.40 ± 0.05	0.39 ± 0.10	0.40 ± 0.04	ns
	SO_4_^2−^ (mg/L)	176.73 ± 61.24	202.71 ± 224.01	40.9 ± 21.85	ns
Sediment	OM (g/kg)	6.31 ± 3.51	4.66 ± 1.94	2.59 ± 0.94	*
	TN (g/kg)	0.29 ± 0.17	0.17 ± 0.06	0.13 ± 0.06	*
	NH_4_^+^-N (mg/kg)	18.24 ± 7.01	17.16 ± 7.05	12.28 ± 3.32	ns
	TP (g/kg)	0.34 ± 0.07	0.48 ± 0.15	0.40 ± 0.11	ns
	AP (g/kg)	5.37 ± 3.79	3.10 ± 2.01	1.90 ± 0.83	*
	Pb (mg/kg)	16.41 ± 1.39	16.89 ± 2.22	17.45 ± 1.33	ns
	Hg (mg/kg)	0.06 ± 0.11	0.03 ± 0.02	0.03 ± 0.02	ns
	As (mg/kg)	8.1 ± 1.10	8.57 ± 1.40	10.17 ± 0.89	**
	Cr (mg/kg)	0.08 ± 0.03	0.09 ± 0.03	0.08 ± 0.03	ns
	Cd (mg/kg)	0.09 ± 0.03	0.24 ± 0.14	0.19 ± 0.14	ns

*, *p* < 0.05; **, *p* < 0.01; ***, *p* < 0.001; ns, non-significant. The means of each environment parameter were averaged value over all sampling sites.

**Table 2:** Key topological properties of microbial networks.

Habitat Type	Nodes	Edges	ACC	APL	Diameter	Density	Modularity
Water	200	1722	0.551	3.138	9	0.087	0.323
Sediment	276	658	0.373	5.531	14	0.017	0.707

ACC, average clustering coefficient. APL, average path length.

## Data Availability

The data presented in this study are available on request from the corresponding author.

## Supplementary Materials

The following supporting information can be downloaded at: https://www.mdpi.com/article/10.3390/microorganisms11020496/s1, Figure S1: Rarefaction curves of bacterial richness of each sample; Figure S2: Relative abundance of different phyla among the three sampling periods. (a) water samples, (b) sediment samples. * *p* < 0.05, ** *p* < 0.01, *** *p* < 0.001, Kruskal-Wallis test; Figure S3: Relative abundances of the dominant bacterial families in water samples. Family with relative abundance < 1% were defined as “other”; Figure S4: Relative abundances of the dominant bacterial families in sediment samples. Family with relative abundance < 1% were defined as “other”; Figure S5: The SPEC-OCCU plots (a) over three sampling periods in water at the family level; Figure S6: The SPEC-OCCU plots (a) over three sampling periods in sediment at the family level; Table S1: Alpha diversity indices of samples based on ASVs; Table S2: Variance inflation factor of the environmental parameters; Table S3: Details of nodes contacted with the drive parameters.
