# Supplementary material for: Determinants and Assembly Mechanism of Bacterial Community Structure in Ningxia Section of the Yellow River
Source: Microorganisms. 2023 Feb 16;11(2):496. doi: 10.3390/microorganisms11020496 (PMC9967387; doi:10.3390/microorganisms11020496)
Supplement: Supplementary file 1 [file microorganisms-11-00496-s001.zip › microorganisms-2198411-Supplementary Figures.pdf]

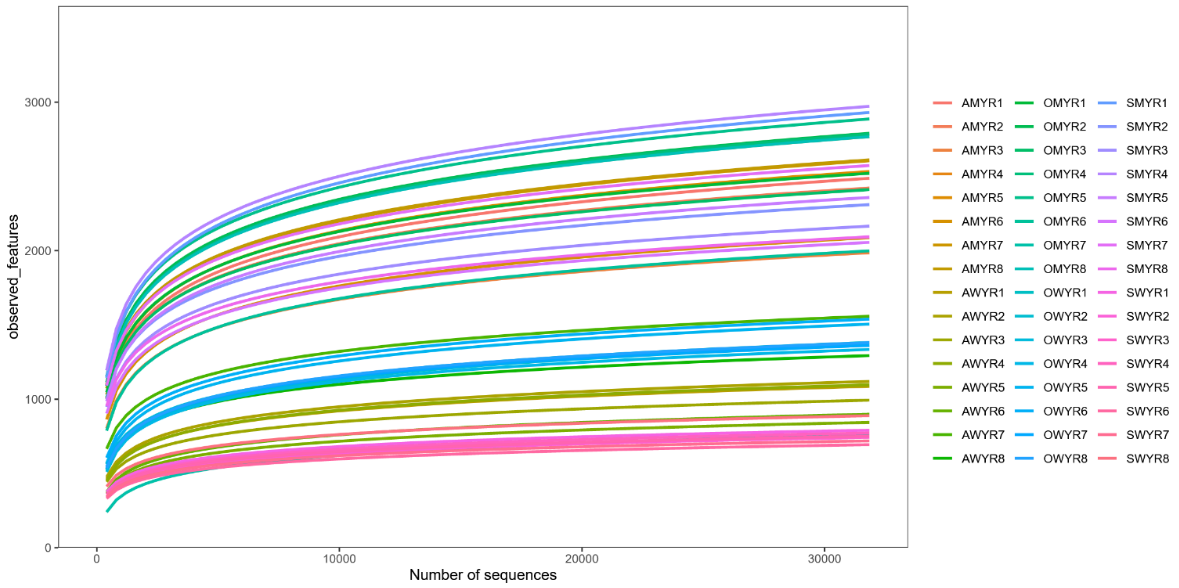

Figure S1. Rarefaction curves of bacterial richness of each sample

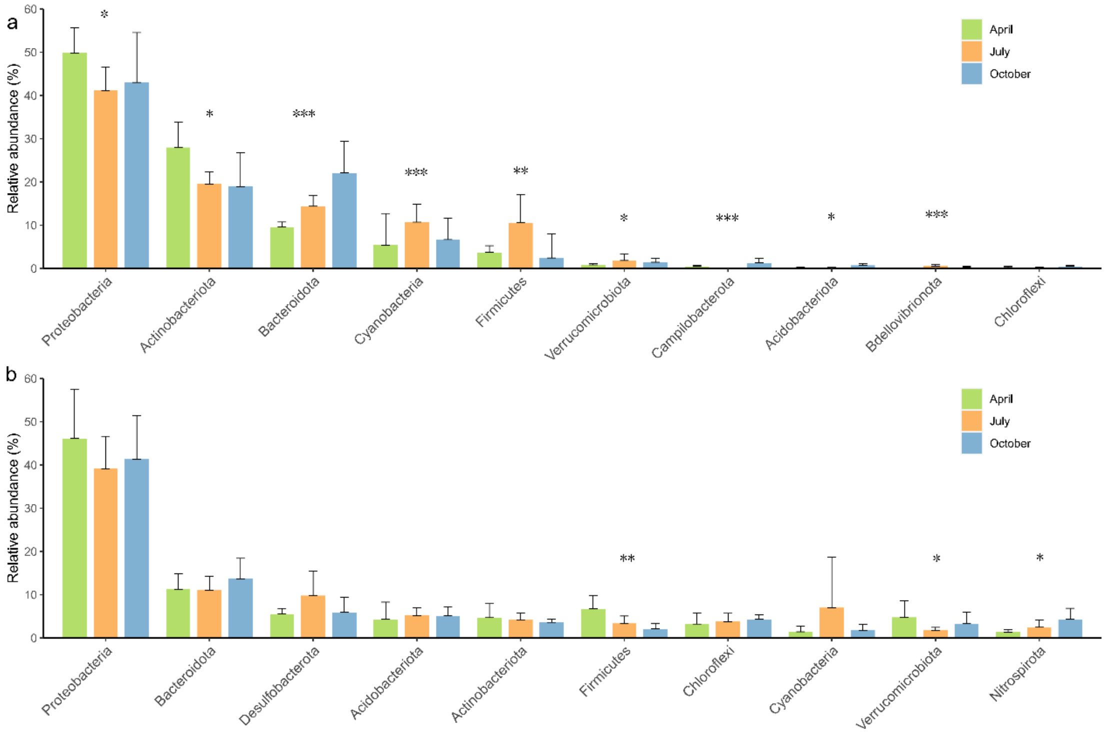

Figure S2. Relative abundance of different phyla among the three sampling periods.(a) water samples, (b)sediment samples. *$p < 0.05$, *** $p < 0.001$, Kruskal-Wallis test.

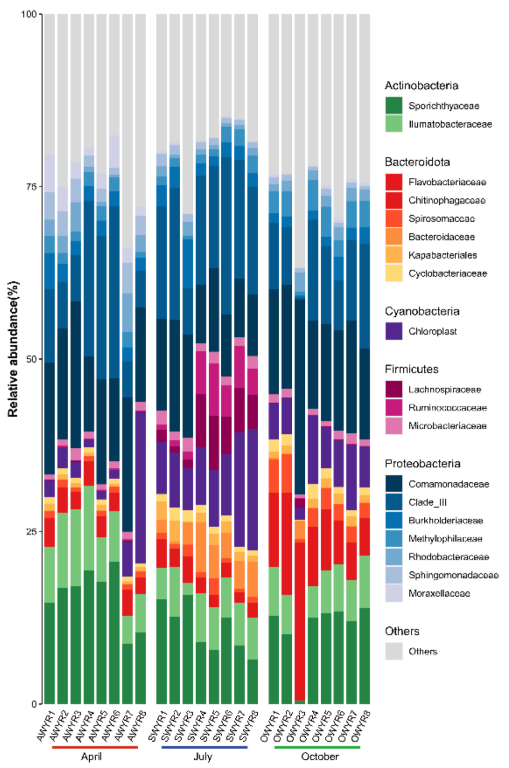

Figure S3. Relative abundances of the dominant bacterial families in water samples. Family with relative abundance <1% were defined as "other".

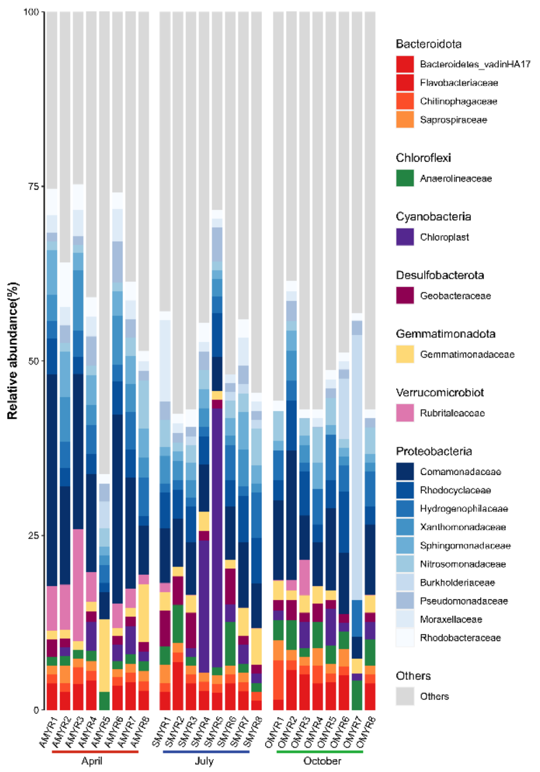

Figure S4. Relative abundances of the dominant bacterial families in sediment samples. Family with relative abundance <1% were defined as "other".

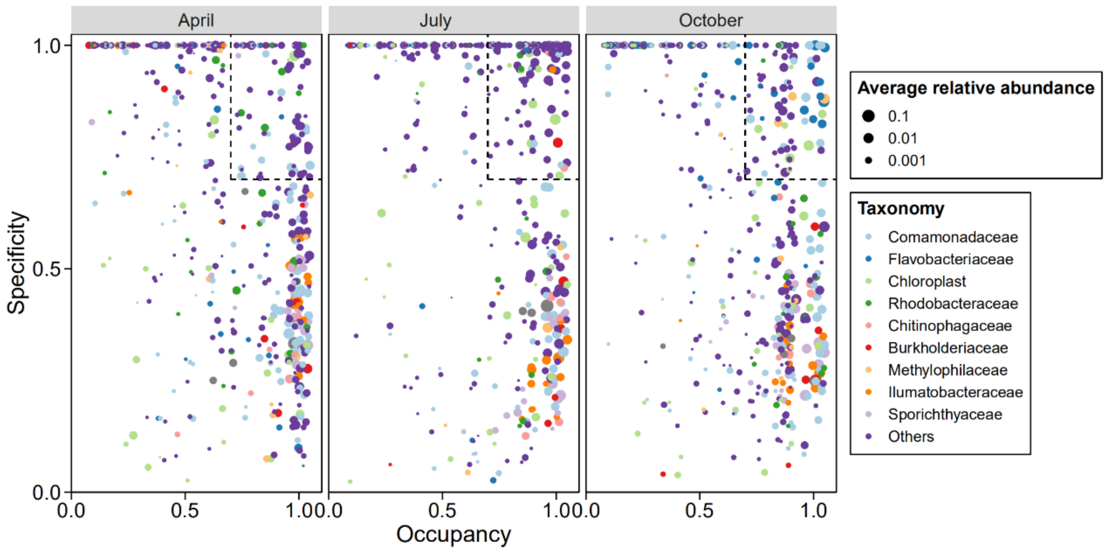

Figure S5. The SPEC-OCCU plots(a) over three sampling periods in water at the family level.

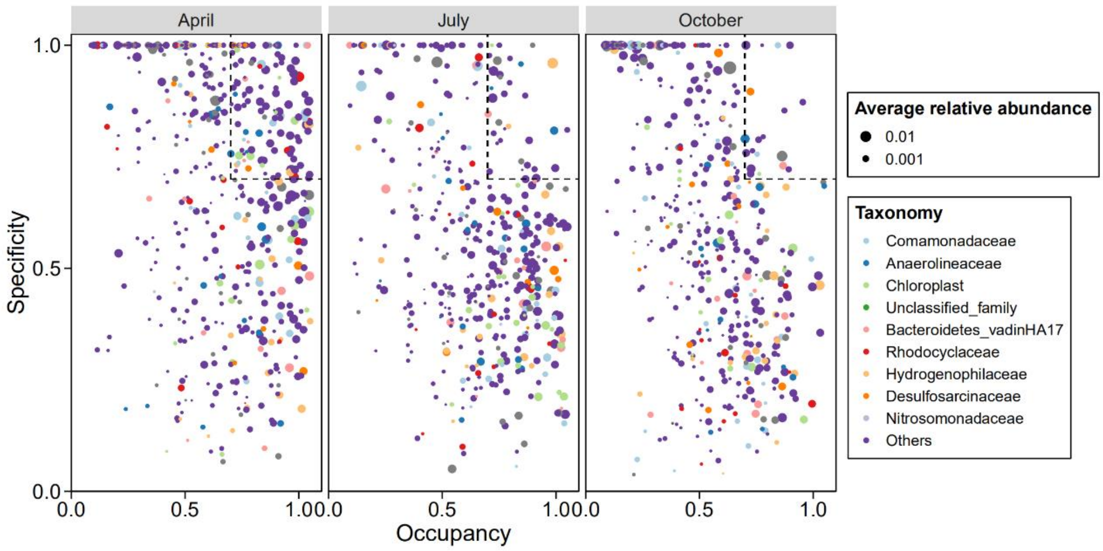

Figure S6. The SPEC-OCCU plots(a) over three sampling periods in sediment at the family level.
